# Supplementary material for: hu.MAP3.0: Atlas of human protein complexes by integration of > 25,000 proteomic experiments
Source: bioRxiv. 2024 Oct 15:2024.10.11.617930. Preprint. [Version 1] doi: 10.1101/2024.10.11.617930 (PMC11507723; doi:10.1101/2024.10.11.617930)
Supplement: 1 — Figure S1. Characterization of hu.MAP 3.0 model performance. (A) Precision-Recall plot evaluated on the leave-out test set of gold standard interactions for different versions of hu.MAP 3.0. The different models were trained with the same literature-curated training set but optimized for different evaluation metrics. The accuracy optimized model shows the greatest performance and is designated as the hu.MAP 3.0 model. (B) Line plot depicting the relationship between precision and the hu.MAP 3.0 model confidence score on the test set interactions, illustrating that increases in confidence score correspond with increased precision. (C) Precision-Recall plot for the hu.MAP 3.0 model on test set interactions, with confidence score thresholds marked as circles, demonstrating clear separation along the curve. (D) Distribution of positive and negative test set interactions across model confidence scores. The histogram highlights the separation of these interactions. Figure S2. Clustering size distribution and annotation enrichment. (A) Distribution of hu.MAP3.0 complex size. Colors in stacked bars represent cluster confidence. (B) Annotation enrichment of hu.MAP3.0 complexes using g:Profiler. Bars represent number of complexes enriched for GO, KEGG, CORUM, Reactome, or Human Phenotype Ontology (HP) annotations that pass a corrected p-value of 0.01. Random represents enriched protein sets when complex membership is shuffled for all complexes. Figure S3. Comparison of ProteomeHD.2, hu.MAP3.0, and OpenCell. (A) Scatterplot of protein pairs’ ProteomeHD.2 covariation score (x-axis) and hu.MAP3.0 confidence score (y-axis). Four quadrants are highlighted where yellow = high in hu.MAP3.0, high in ProteomeHD.2, blue = high in hu.MAP3.0, low in ProteomeHD.2, orange = low in hu.MAP3.0, high in ProteomeHD.2, and purple = low in both. (B) Comparison of four quadrants in (A) to an orthogonal protein interaction dataset, OpenCell, not included in either hu.MAP3.0 nor ProteomeHD.2. Bars r [file NIHPP2024.10.11.617930V1-supplement-1.pdf]

Figure S1

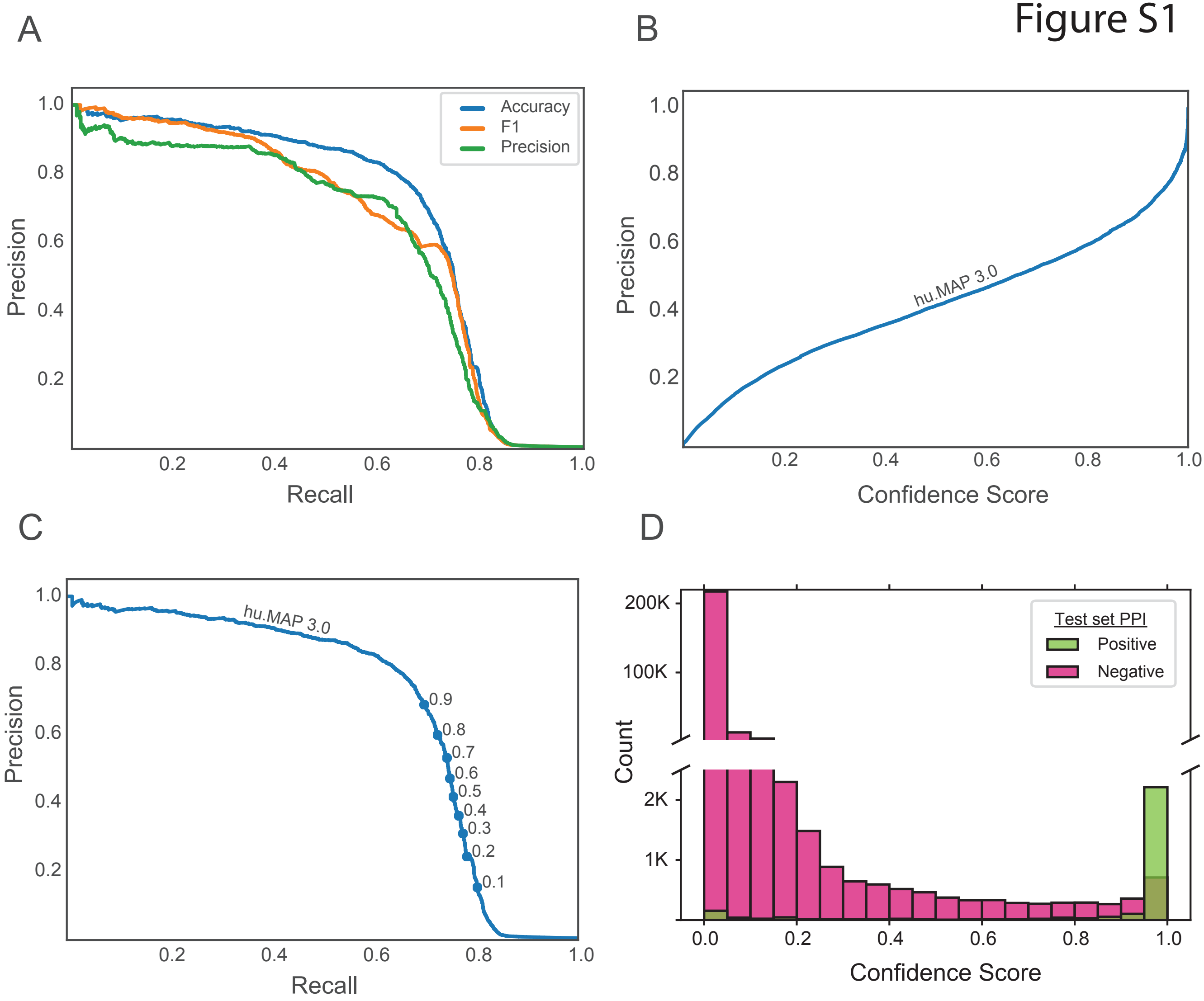

A
Accuracy
F1
Precision
1.0
0.8
0.6
0.4
0.2
Precision
0.2
0.4
0.6
0.8
1.0
Recall
B
1.0
0.8
0.6
0.4
0.2
Precision
hu.MAP 3.0
0.2
0.4
0.6
0.8
1.0
Confidence Score
C
1.0
0.8
0.6
0.4
0.2
Precision
hu.MAP 3.0
0.9
0.8
0.7
0.6
0.5
0.4
0.3
0.2
0.1
0.2
0.4
0.6
0.8
1.0
Recall
D
200K
100K
2K
1K
Count
Test set PPI
Positive
Negative
0.0
0.2
0.4
0.6
0.8
1.0
Confidence Score

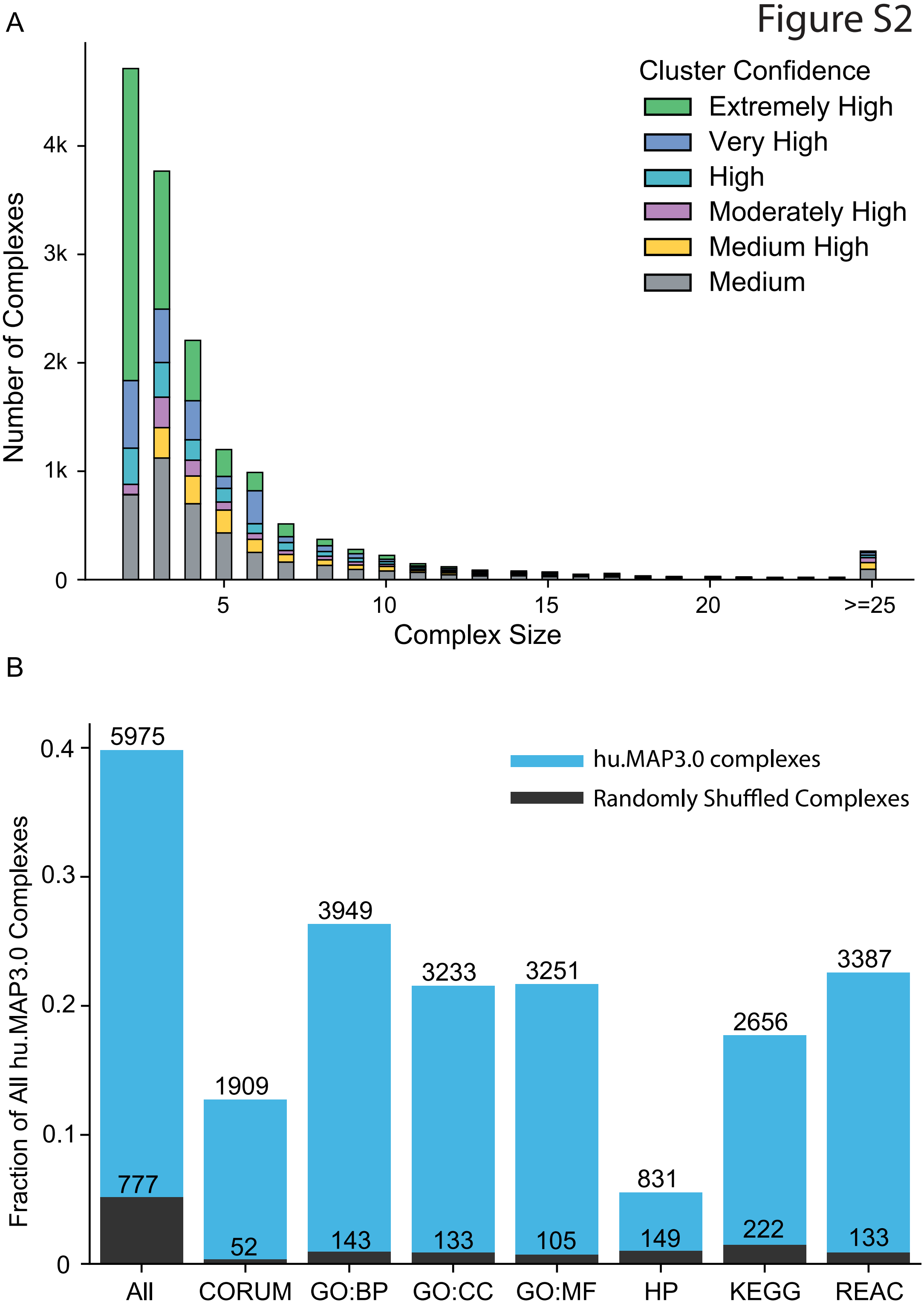
Figure S2
A
Cluster Confidence
Extremely High
Very High
High
Moderately High
Medium High
Medium
Number of Complexes
0
1k
2k
3k
4k
5
10
15
20
>=25
Complex Size
B
hu.MAP3.0 complexes
Randomly Shuffled Complexes
Fraction of All hu.MAP3.0 Complexes
0
0.1
0.2
0.3
0.4
5975
777
1909
52
3949
143
3233
133
3251
105
831
149
2656
222
3387
133
All
CORUM
GO:BP
GO:CC
GO:MF
HP
KEGG
REAC

Figure S3

bioRxiv preprint doi: https://doi.org/10.1101/2024.10.11.617930; this version posted October 15, 2024. The copyright holder for this preprint (which was not certified by peer review) is the author/funder, who has granted bioRxiv a license to display the preprint in perpetuity. It is made available under aCC-BY 4.0 International license.

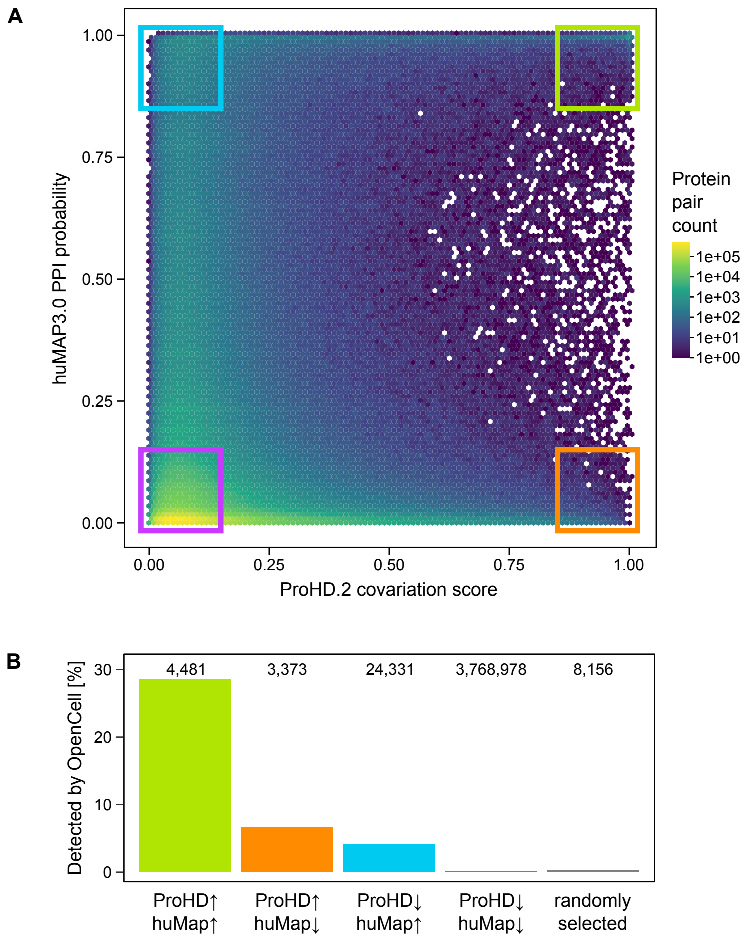

Figure S4

bioRxiv preprint doi: https://doi.org/10.1101/2024.10.11.617930; this version posted October 15, 2024. The copyright holder for this preprint (which was not certified by peer review) is the author/funder, who has granted bioRxiv a license to display the preprint in perpetuity. It is made available under aCC-BY 4.0 International license.

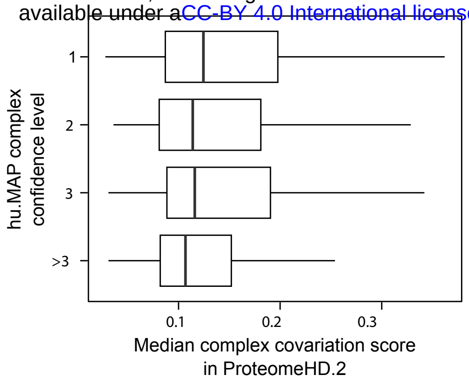

Figure S5

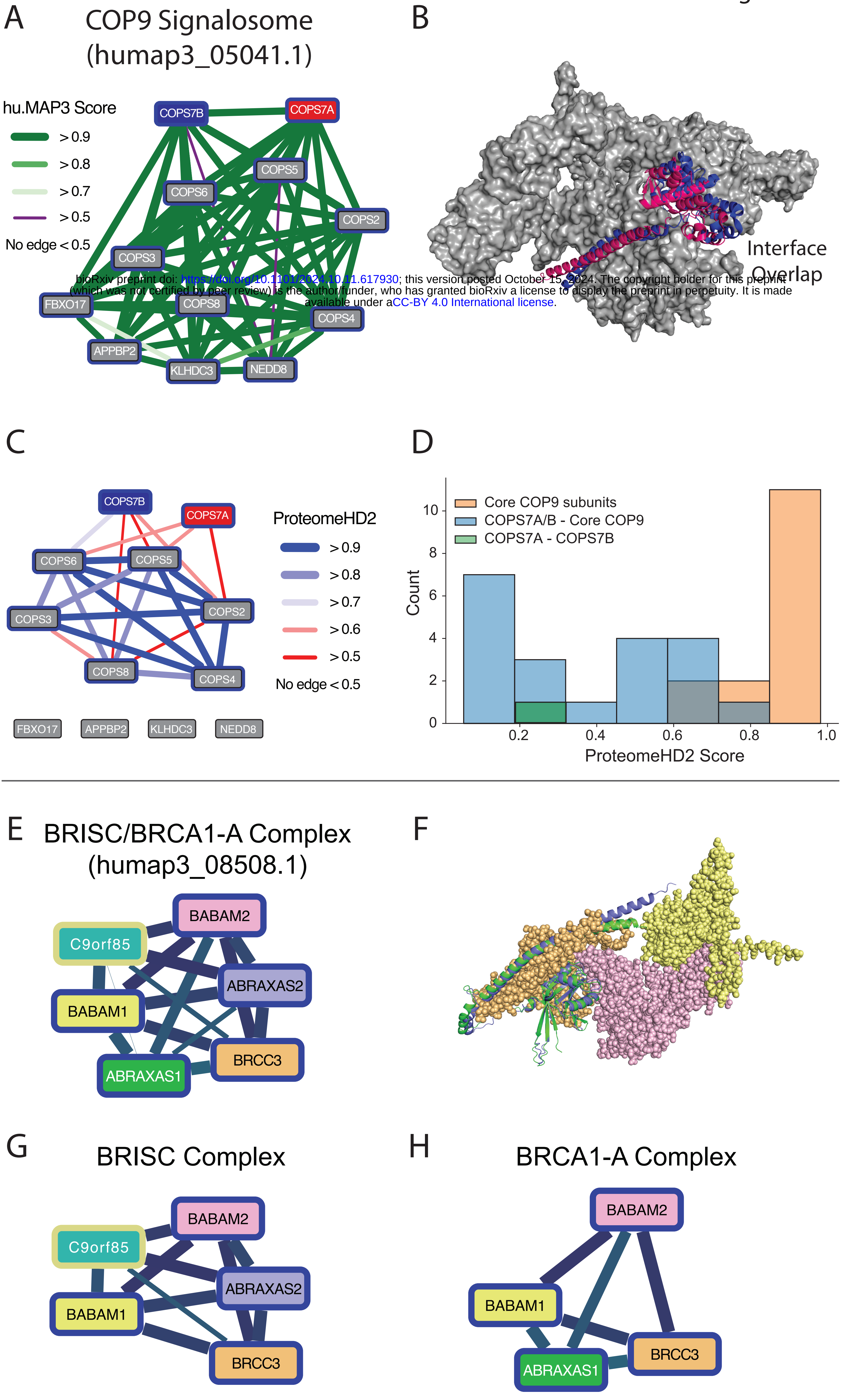

bioRxiv preprint doi: https://doi.org/10.1101/2024.10.11.617930; this version posted October 15, 2024. The copyright holder for this preprint (which was not certified by peer review) is the author/funder, who has granted bioRxiv a license to display the preprint in perpetuity. It is made available under aCC-BY 4.0 International license.

Figure S6

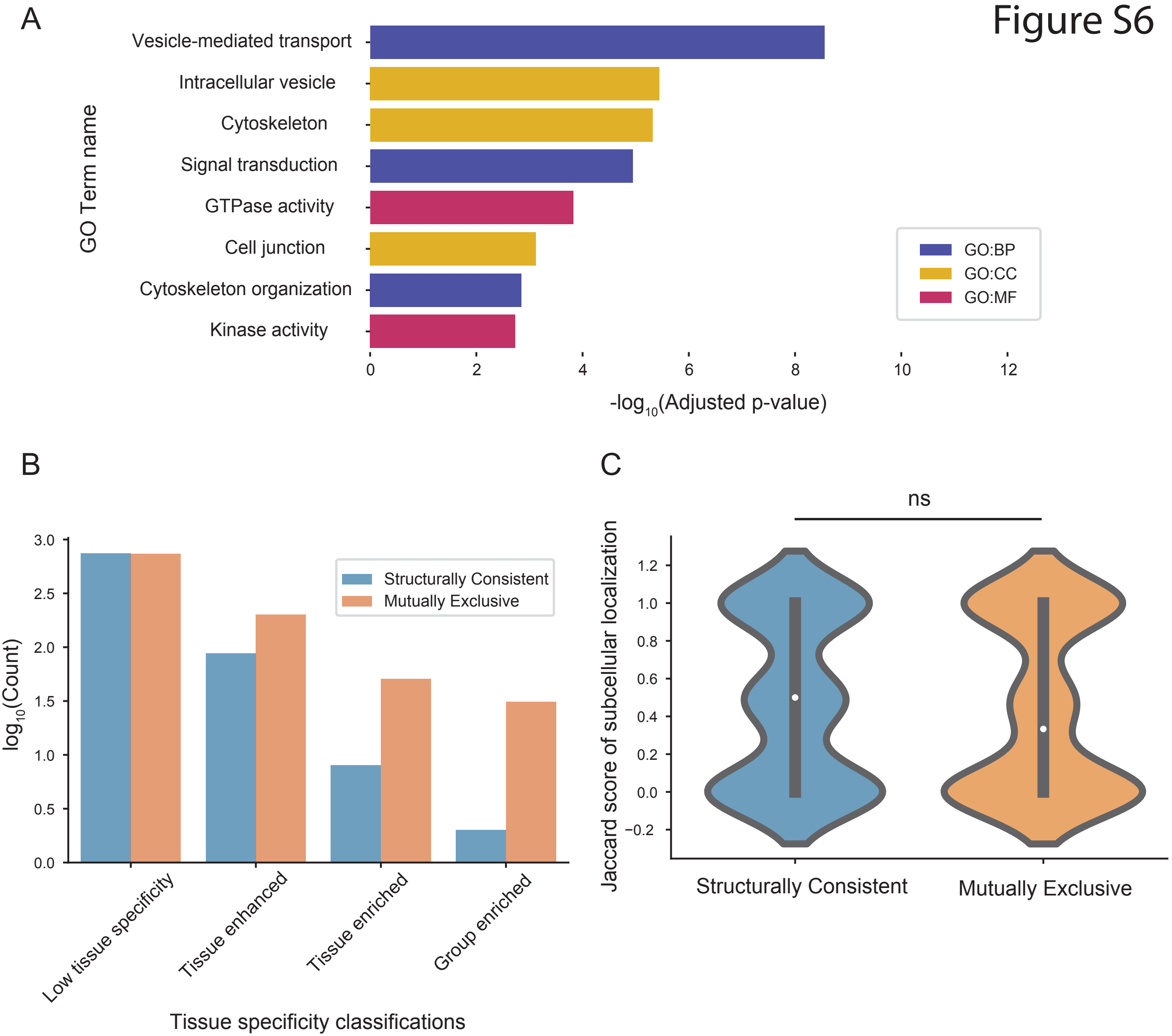
A
Vesicle-mediated transport
Intracellular vesicle
Cytoskeleton
Signal transduction
GTPase activity
Cell junction
Cytoskeleton organization
Kinase activity
GO Term name
GO:BP
GO:CC
GO:MF
0
2
4
6
8
10
12
-log10(Adjusted p-value)
B
Structurally Consistent
Mutually Exclusive
log10(Count)
3.0
2.5
2.0
1.5
1.0
0.5
0.0
Low tissue specificity
Tissue enhanced
Tissue enriched
Group enriched
Tissue specificity classifications
C
ns
Jaccard score of subcellular localization
1.2
1.0
0.8
0.6
0.4
0.2
0.0
−0.2
Structurally Consistent
Mutually Exclusive

Figure S7

bioRxiv preprint doi: https://doi.org/10.1101/2024.10.11.617930; this version posted October 15, 2024. The copyright holder for this preprint (which was not certified by peer review) is the author/funder, who has granted bioRxiv a license to display the preprint in perpetuity. It is made available under aCC-BY 4.0 International license.

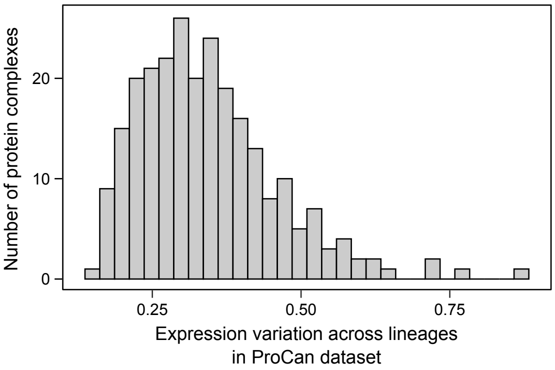

Figure S8

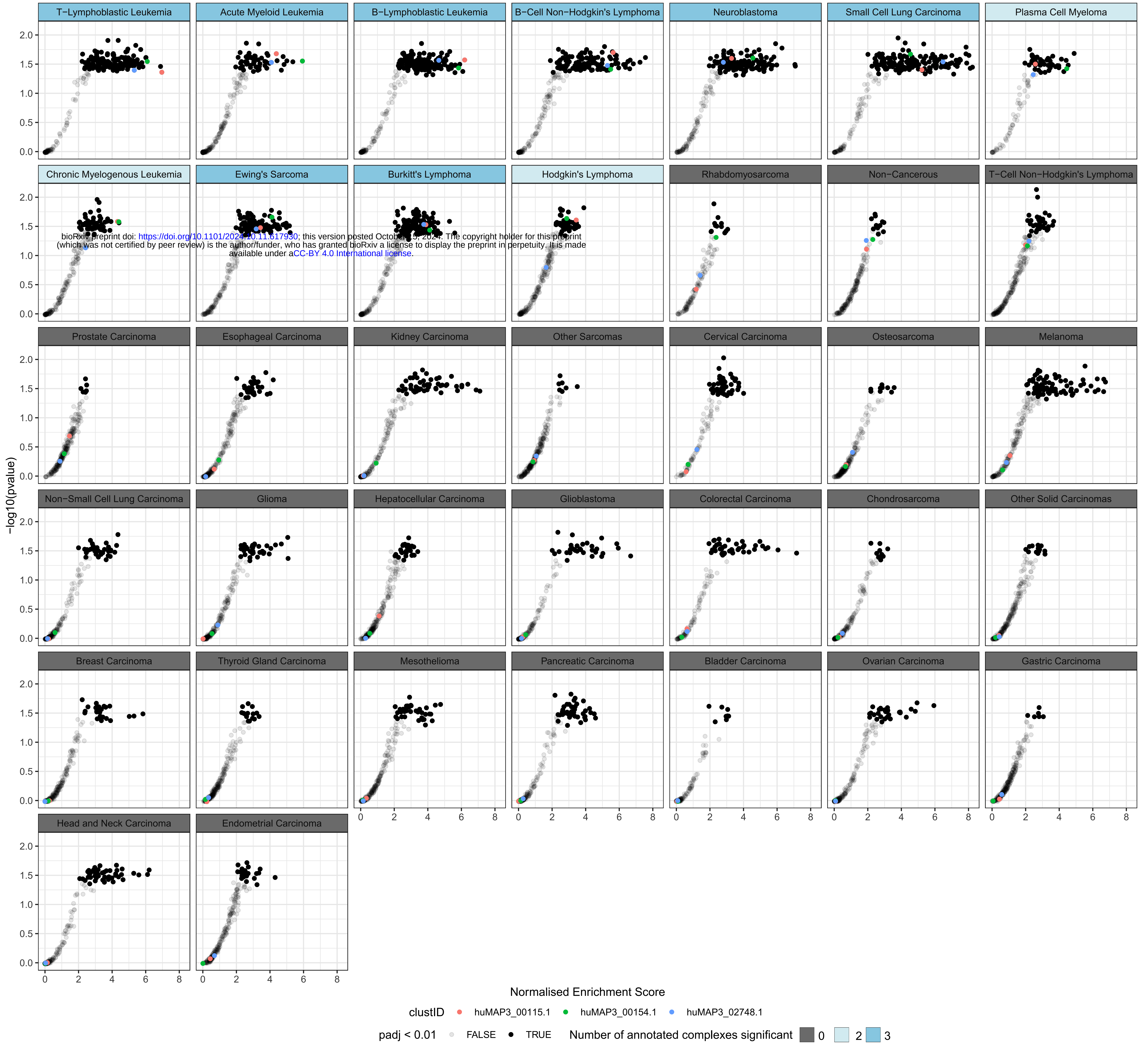

bioRxiv preprint doi: https://doi.org/10.1101/2024.10.11.617930; this version posted October 15, 2024. The copyright holder for this preprint (which was not certified by peer review) is the author/funder, who has granted bioRxiv a license to display the preprint in perpetuity. It is made available under aCC-BY 4.0 International license.
